# Dysregulated Levels of Circulating Autoantibodies against Neuronal and Nervous System Autoantigens in COVID-19 Patients

**DOI:** 10.3390/diagnostics13040687

**Published:** 2023-02-12

**Authors:** Yael Lavi, Aristo Vojdani, Gilad Halpert, Kassem Sharif, Yuri Ostrinski, Israel Zyskind, Miriam T Lattin, Jason Zimmerman, Jonathan I Silverberg, Avi Z Rosenberg, Yehuda Shoenfeld, Howard Amital

**Affiliations:** 1 Sackler Faculty of Medicine, Tel Aviv University, Tel Aviv 69978, Israel; 2 Immunosciences Lab, Inc., Los Angeles, CA 90035, USA; 3 Cyrex Laboratories, LLC, Phoenix, AZ 85034, USA; 4 Zabludowicz Center for Autoimmune Diseases, Sheba Medical Center, Affiliated with the Sackler Faculty of Medicine, Tel Aviv University, Tel Aviv 69978, Israel; 5 Department of Molecular Biology, Ariel University, Ariel 40700, Israel; 6 Department of Medicine B, Sheba Medical Center, Tel Hashomer, Ramat Gan 52621, Israel; 7 Department of Pediatrics, NYU Langone Medical Center, New York, NY 10016, USA; 8 Maimonides Medical Center, Brooklyn, NY 11219, USA; 9 Department of Biology, Yeshiva University, New York, NY 10461, USA; 10 Department of Dermatology, George Washington University School of Medicine and Health Sciences, Washington, DC 20052, USA; 11 Department of Pathology, Johns Hopkins University, Baltimore, MD 21205, USA

**Keywords:** autoantibodies, central nervous system, autoimmunity, SARS-CoV-2, COVID-19, autoantigens

## Abstract

Background: COVID-19 is a heterogenous disease resulting in long-term sequela in predisposed individuals. It is not uncommon that recovering patients endure non-respiratory ill-defined manifestations, including anosmia, and neurological and cognitive deficit persisting beyond recovery—a constellation of conditions that are grouped under the umbrella of long-term COVID-19 syndrome. Association between COVID-19 and autoimmune responses in predisposed individuals was shown in several studies. Aim and methods: To investigate autoimmune responses against neuronal and CNS autoantigens in SARS-CoV-2-infected patients, we performed a cross-sectional study with 246 participants, including 169 COVID-19 patients and 77 controls. Levels of antibodies against the acetylcholine receptor, glutamate receptor, amyloid β peptide, alpha-synucleins, dopamine 1 receptor, dopamine 2 receptor, tau protein, GAD-65, N-methyl D-aspartate (NMDA) receptor, BDNF, cerebellar, ganglioside, myelin basic protein, myelin oligodendrocyte glycoprotein, S100-B, glial fibrillary acidic protein, and enteric nerve were measured using an Enzyme-Linked Immunosorbent Assay (ELISA). Circulating levels of autoantibodies were compared between healthy controls and COVID-19 patients and then classified by disease severity (mild [*n* = 74], severe [*n* = 65], and requiring supplemental oxygen [*n* = 32]). Results: COVID-19 patients were found to have dysregulated autoantibody levels correlating with the disease severity, e.g., IgG to dopamine 1 receptor, NMDA receptors, brain-derived neurotrophic factor, and myelin oligodendrocyte glycoprotein. Elevated levels of IgA autoantibodies against amyloid β peptide, acetylcholine receptor, dopamine 2 receptor, myelin basic protein, and α-synuclein were detected in COVID-19 patients compared with healthy controls. Lower IgA autoantibody levels against NMDA receptors, and IgG autoantibodies against glutamic acid decarboxylase 65, amyloid β peptide, tau protein, enteric nerve, and S100-B were detected in COVID-19 patients versus healthy controls. Some of these antibodies have known clinical correlations with symptoms commonly reported in the long COVID-19 syndrome. Conclusions: Overall, our study shows a widespread dysregulation in the titer of various autoantibodies against neuronal and CNS-related autoantigens in convalescent COVID-19 patients. Further research is needed to provide insight into the association between these neuronal autoantibodies and the enigmatic neurological and psychological symptoms reported in COVID-19 patients.

## 1. Introduction

The coronavirus disease 2019 (COVID-19) started in Wuhan, the capital of Hubei Province in the People’s Republic of China, and was declared a pandemic in early March 2020. COVID-19 is caused by a coronavirus variant named severe acute respiratory syndrome-like coronavirus (SARS-CoV-2). Increasing evidence points towards an association between COVID-19 and the development of autoimmune responses [1,2,3] in predisposed individuals, including dysregulation of autoantibodies levels—e.g., anti-double-stranded DNA (Anti-dsDNA) [4], type-I interferons [5], anti-prothrombin, anti-heparin PF4, anti-IFNs, P-ANCA, RF, anti β2 glycoprotein I, among others [2]. Mechanisms explaining these responses include molecular mimicry, immune overstimulation, epitope spreading, and the presentation of cryptic antigens [3]. Molecular mimicry is one of the more dominant mechanisms attributed to the pathological conditions of autoimmune diseases; this occurs when bacterial or viral antigens have cross-reactive self-antigens. This cross-reactivity has been shown as a probable mechanism in several autoimmune diseases associated with specific self-antigens [6].

Typical COVID-19 symptoms include anosmia (loss of sense of smell) and ageusia (impaired sense of taste) reported in 60% of patients, suggesting an involvement of the nervous system [7]. Neurotropism of SARS-CoV-2 provides an explanation for a wide spectrum of neuropsychiatric symptoms persisting beyond recuperation [8]. Neurologic symptoms relating to the peripheral nervous system include anosmia, ageusia, acute myelitis, Guillain-Barré Syndrome, Miller-Fisher syndrome, and polyneuritis cranialis. There is conflicting evidence on whether the virus directly infects the CNS. While few studies showed viral detection in the nervous system such as in the cerebrospinal fluid of a COVID-19 patient with aseptic encephalitis, other studies showed viral detection in non-CNS-infected organs [8]. 

A higher prevalence of autoantibodies produced against the autonomic nervous system receptors was noted in subjects with long-COVID-19 symptoms [9]. Several studies reported the reaction of both animal and human monoclonal antibodies against SARS-CoV-2 spike and nucleoprotein with different nervous system antigens, including β-amyloid peptide, synapsin, neurofilament protein, myelin basic protein, S100B, glutamic acid decarboxylase, and mitochondrial M antigen [10,11]. 

Autoantibodies are well-recognized to cause multiple diseases of the nervous system—e.g., antibodies against the post-synaptic acetylcholine receptor in myasthenia gravis, amyloid- ß in neurodegenerative disorders [12,13], and NMDA receptors in neuropsychiatric diseases in SLE patients [14]. Studies also showed dysregulation in the titer and functional activity of non-classical autoantibodies directed against G-protein-coupled receptors (GPCRs) of the autonomic nervous system, which might play a role in suspected autoimmune dysautonomic-related disorders—e.g., chronic fatigue syndrome, postural orthostatic tachycardia, complex regional pain syndrome, and silicone implant incompatibility syndrome [15]. There are also several neurologic disorders with a known correlation to former viral or bacterial infections, such as Guillain-Barré syndrome, autoimmune encephalitis, myasthenia gravis, and Stiff person syndrome [16]. Our group previously found dysregulation in the level of a wide range of autoantibodies directed against diverse autoantigens in convalescing COVID-19 patients [17]. (A descriptive list of these antigens can be found in Supplementary Table S1). 

In the current study, we focus on the potential changes in the production of autoantibodies against the nervous system aiming to uncover clues explaining enigmatic COVID-19 symptoms.

## 2. Results

Two hundred and forty-six adults were recruited from Jewish communities across five states of the United States of America to participate in the Multi-Institutional Study Analyzing Anti–CoV-2 Antibodies (MITZVA) cohort. Convalescing COVID-19 patients were categorized according to the clinical severity of COVID-19 based on self-reported symptoms.

Dysregulated serum levels of autoantibodies against the amyloid β peptide (IgA and IgG), NMDA receptor (IgA and IgG), GAD-65 (IgG), S-100B (IgG), BDNF (IgG), D1 receptor (IgG), tau protein (IgG), acetylcholine receptor (IgA), α-synucleins (IgA), myelin basic protein (IgA), etc. were detected in patients with COVID-19 compared with healthy controls (Table 1). The full results are detailed in Supplementary Table S2.

Several antibodies were elevated in the sera of patients with past COVID-19 infections regardless of disease severity. Levels of antibodies against the acetylcholine receptor (IgA), α-synucleins (IgA), amyloid β peptide (IgA), D2 receptor (IgA), and myelin basic protein (IgA) were elevated in COVID-19 patients’ sera regardless of disease severity. In contrast, levels of antibodies against tau protein (IgG), S100B (IgG), NMDA receptor (IgA), enteric nerve (IgG), amyloid β peptide (IgG), and GAD-65 (IgG) were lower in the sera of COVID-19 patients.

Antibodies against NMDA receptors (IgG), BDNF (IgG), GAD-65 (IgG), D1 receptor (IgG), and myelin oligodendrocyte glycoprotein (IgG) were elevated in patients with severe COVID-19 and those who required supplemental oxygen compared with those with mild COVID-19.

Several autoantibodies did not differ significantly between COVID-19 severity compared with controls, including glial fibrillary acidic protein (IgA/IgG) and glutamate receptor (IgA/IgG).

Antibody levels were further analyzed for inherent correlations between their levels (Table 2). No correlations were found between IgA antibodies and IgG antibodies. While widespread strong correlations were observed between all IgA antibody groups, correlations between different IgG antibodies were also observed. Several IgG antibodies did not show strong correlations with other IgG antibodies and are apparently relatively specific, such as IgG antibodies against the BDNF, myelin basic protein, NMDA receptor, and S100-B. 

## 3. Discussion

Our study showed that levels of several autoantibodies to nervous system epitopes were associated with the clinical severity of COVID-19, including IgG antibodies to the dopamine 1 receptor, NMDA receptors, brain-derived neurotrophic factor (BNDF), and myelin oligodendrocyte glycoprotein. Dopamine 1 receptors (D1R) are highly expressed on central nervous system neurons and affect cognition, emotion, locomotor activity, regulation of hunger, satiety, and endocrine system—factors that are associated with neuropsychiatric disorders [18,19,20]. Therefore, we hypothesize that the overproduction of antibodies directed against D1R, as can be seen in our cohort (Table 2), might interfere with dopamine binding to its receptor or directly affect D1R signaling pathways and, thus, contribute to cognitive and emotional disturbances reported in COVID-19 patients. NMDA receptors are critical for synaptic plasticity, which is integral in mediating learning and memory formation [21]. We found elevated levels of IgG antibodies against the NMDA receptor in severe COVID-19 patients (Table 1). Our results are in correlation with recent observations indicating antibodies against NMDA receptors in the cerebrospinal fluid of eight COVID-19 patients suffering from autoimmune encephalitis [22]. BNDF is important for long-term memory formation [23] and is associated with the pathogenesis of several psychiatric conditions [24,25]. In our cohort, we showed increased levels of IgG antibodies against BDNF in severe COVID-19 patients and in those who required supplemental oxygen (Table 1). Muscle aches and deconditioning associated with severe COVID-19 presentation potentially result in the extravasation of the BDNF from skeletal muscles and, subsequently, result in epitope spreading, leaving patients at a higher risk of developing autoantibodies against the BDNF. Antibodies against myelin oligodendrocyte glycoprotein are associated with inflammatory demyelinating diseases contributing to acute myelitis in COVID-19 patients and increasing the risk of developing a chronic or recurrent demyelinating disease in convalescing patients [26]. These findings are in line with our data, which describe a significant production of IgG antibodies against myelin oligodendrocyte glycoprotein in COVID-19 patients who required supplemental oxygen as compared with healthy controls (Table 1). The association between the production of autoantibodies and COVID-19 severity may provide an explanation to the unclear pathogenesis of long-lasting cognitive and psychiatric symptoms reported with COVID-19. 

Our study also found several antibodies that were elevated in the sera of COVID-19 patients regardless of disease severity, including the acetylcholine receptor (IgA), α-synucleins (IgA), amyloid β peptide (IgA), dopamine 2 (D2) receptor (IgA), and myelin basic protein (IgA) (Table 1). α-Synuclein protein is reported to be linked neuropathologically to Parkinson’s disease. It is possible that autoantibodies interfering with this protein could provide insight into the pathogenesis of Parkinson-like manifestations [27] reported in some post-COVID-19 patients. Autoantibodies against the D2 receptors are mostly known for their possible role in the pathogenesis of basal ganglia encephalitis [28] presenting with dystonia, oculogyric crisis, chorea, parkinsonism, and different neuropsychiatric manifestations [28,29]. Elevated levels of IgA antibodies against dopamine 2 receptors were observed in COVID-19 patients regardless of severity of the clinical disease, which might be explained by the chemotaxis of lymphocytes to the target organs, and respiratory and digestive systems, which are abundant with diverse IgA antibodies. SARS-CoV-2 mainly targets the respiratory system and enterocytes [30], both abundantly secreting IgA. Levels of IgA autoantibodies may be elevated as a physiologic response to the COVID-19 infection and may not be secondary to an underlying autoimmune inflammatory response, probably explaining elevated levels of autoantibodies regardless of the clinical severity of COVID-19. Notably, the observed elevation of IgA in severe COVID-19 patients could be secondary to the natural abundance of IgA levels in both the respiratory and gastrointestinal systems; however, the possibility of cross-reactivity between IgA antibodies should be taken into consideration [31].

We also observed lower levels of autoantibodies against NMDA receptors (IgA), glutamic acid decarboxylase 65 (IgG), amyloid β peptide (IgG), tau protein (IgG), enteric nerve (IgG), and S100-B (IgG) in COVID-19 patients compared with healthy controls (Table 1). Several of these autoantibody levels returned to normal upon recovery from a severe disease, a finding that might be explained by the increased expression of tissue autoantigens leading to autoantibody consumption in tissues and, thus, resulting in a decline of autoantibody titers in the sera. We hypothesized that in severe COVID-19 cases, cells undergo apoptosis either as a response to viral infection or secondary to the inflammatory process, resulting in intracellular antigen exposure, thereby stimulating autoantibody production. Another explanation for the lower autoantibody levels may be due to increased levels of antigen-bound autoantibodies and the formation of immune complexes supported by findings from a study by Maftei et al. [32], reporting significantly higher levels of antigen-IgG immune complexes in Alzheimer’s disease patients, the levels of which correlated negatively with clinical symptoms. Levels of antibodies against tau protein are lower in Alzheimer’s patients [33]. Our study found lower levels of IgG autoantibodies against the tau protein in the sera of COVID-19 patients. This may hint at a possible rise in the prevalence of Alzheimer’s dementia in convalescing COVID-19 patients. S100-B is considered a marker of glial or ganglial cell damage. Levels of IgG antibodies against S100-B were significantly lower in COVID-19 patients regardless of disease severity when compared with the healthy control group, which could be explained by tissue consumption of autoantibodies. Another explanation for the difference in the levels of these antibodies is the possibility that S100-B autoantibodies represent a part of the natural homeostatic immune system, thereby rendering elevated levels of S100-B autoantibodies as a possible predictor of immune competence and a protecting factor against severe COVID-19.

Our study is a cross-sectional prevalence study with limitations inherent to the study design. Our study does not include levels of autoantibodies before developing COVID-19, thus, precluding our ability to determine whether the dysregulated levels of autoantibodies were present beforehand. Our study quantified serum levels of autoantibodies, but functional activity and tissue levels of these autoantibodies were not investigated. Further studies should ascertain whether these autoantibodies possess clinical significance and a potential target for intervention. It is important to emphasize that this study addresses the possible influence of COVID-19 illness and severity on the production of neuronal autoantibodies and does not assess their direct correlation to neuronal symptoms. Another main limitation of our study is the lack of congruence between our severity scale classification and the well-accepted WHO severity classification. Finally, data regarding disease severity was based on self-reported symptoms and did not include the necessary data to allow classification based on WHO severity classification.

In conclusion, our study shows widespread dysregulation in the titer of various autoantibodies against neuronal and CNS-related autoantigens in convalescent COVID-19 patients. Further research is needed to provide insight into the association between these neuronal autoantibodies and the enigmatic neurological and psychological symptoms reported in COVID-19 patients. 

## 4. Methods

### 4.1. Patient Recruitment

Included in this study were 246 adults from Jewish communities across 5 states of the United States of America that participated in the Multi-Institutional Study Analyzing Anti–CoV-2 Antibodies (MITZVA) cohort. Recruitment of study participants was organized in partnership with local nonprofit and social service organizations offering antibody testing to symptomatic or asymptomatic adults within the large orthodox Jewish communities [34]. 

Healthy controls were defined as subjects with no detected antibodies against SARS-CoV-2. A hundred and sixty-nine individuals were positive for SARS-CoV-2 by PCR and antibodies and were classified into one of three groups according to clinical presentation: mild (*n* = 74), severe (*n* = 63), and oxygen-dependent (*n* = 32) groups. The demographic characteristics of the groups can be found in Table 3.

Disease severity for SARS-CoV-2-positive individuals was based on self-reported symptoms. Severe cases were defined as patients with fever lasting more than seven days, maximal body temperature greater than or equal to 102 Fahrenheit (F), and at least one other COVID-19 symptom (fever, muscle aches, anosmia, dysgeusia, headache, diarrhea, vomiting, stomach ache, or rash). Patients treated with oxygen were classified as oxygen-dependent.

In contrast, patients with fever lasting less than or equal to one day, maximal body temperature of <100 °F (37.8 °C), and at least one other COVID-19 symptom were classified as mild cases. It is noteworthy that all the subjects included in this study fit the definition of only one of the three categories. Age- and sex-matched controls were randomly selected and were defined as those with no COVID-19 symptoms or SARS-CoV-2 IgM/IgG antibodies. The demographic data were compared to assess homogeneity (see Table 3).

The study design and research protocol were approved by the IntegReview institutional review board. Signed electronic informed consent was obtained from all participants. This study followed the Strengthening the Reporting of Observational Studies in Epidemiology (STROBE) reporting guidelines [34].

### 4.2. Detection of Autoantibodies

All sera were tested for IgG anti-SARS-CoV-2 antibody using a Zeus Scientific kit measuring levels of IgG antibodies for both spike and nucleoprotein. Using this kit, only one sample from the control sera tested positive and two samples from COVID-19 group tested negative for IgG antibody.

### 4.3. Antigens

Acetylcholine receptor a-subunit R-97-116, dopamine D_1_ receptor domain–3, 4 and 5, dopamine D_2_ receptor DRD2 E1.1 and E1.2, *N*-methyl-D-aspartate receptors NR2A and NR2B, amyloid-b peptide 1-42, and enteric nerve ribonuclear polypeptide A were synthesized by Bio-Synthesis (Lewisville, TX, USA), and tau protein recombinant brain-derived neurotropic factor (BDNF) was purchased from R&D Systems (Minneapolis, MN, USA).

### 4.4. Measurement of IgG and IgA against Different Antigens

Acetylcholine-R, D_1_, D_2_, NMDA-R, Ab-42 peptide, tau protein, enteric nerve antigen, and BDNF at a concentration of 1 mg/mL were prepared in 0.01 M phosphate buffer saline (PBS) pH 7.4. One hundred µL of these antigens at the optimal concentration ranging from 10–20 microgram/mL or 1–2 micrograms per well, prepared in 0.01 M PBS pH 7.4, were added to a series of microtiter plates. Several wells were also coated with 2% bovine serum albumin (BSA) or human serum albumin (HSA) and used as controls. The ELISA plates were incubated overnight at 4 °C and then were washed five times with 250 µL of 0.01 M PBS containing 0.05% Tween 20 pH 7.4.

The non-specific binding of immunoglobins was prevented by adding of 200 µL of 2% BSA in PBS, which was then incubated overnight at 4 °C. Plates were washed as described above, and then serum samples from controls and the SARS-CoV-2 groups diluted at 1:50 for the determination of IgA antibody, and 1:100 for the determination of IgG antibody in serum diluent buffer or 1% BSA in PBS containing 0.05% Tween 20 were added to the wells of ELISA plates and incubated for one hour at room temperature. Sera from patients with myasthenia gravis, Sydenham’s chorea, neuropsychiatric disorder, Alzheimer’s disease, and irritable bowel syndrome (IBS) at different dilutions were used as calibrators and positive controls, and for the calculation of ELISA indices.

Plates were washed, and then alkaline phosphatase goat anti-human IgG or IgA F(ab,)2 fragments (KPI, Gaithesburg, MD, USA) at an optimal dilution of 1:200 for IgA and 1:600 for IgG in 1% BSA-PBS were added to each appropriate well. The plates were then incubated for another hour at room temperature. After five washes with PBS-Tween buffer, the enzyme reaction was started by adding 100 µL of paranitrophenylphosphate at a concentration of 1 mg/mL in diethanolamine buffer containing 1 mM MgCl2 and sodium azide pH 9.8. Forty-five minutes later, the reaction was stopped with 50 µL of 1 N NaOH. The optical density (OD) was read at 405 nm with a microtiter plate reader. The ODs of the control wells containing HSA or BSA which were not higher than 0.15 were subtracted from all other wells to exclude non-specific binding.

The ELISA index for each antibody was calculated based on the following formula:

$$\mathrm{Antibody}\;\mathrm{ELISA}\;\mathrm{index}\;=\frac{\mathrm{OD}\;\mathrm{of}\;\mathrm{sample}\;-\;\mathrm{OD}\;\mathrm{of}\;\mathrm{negative}\;\mathrm{control}}{\mathrm{OD}\;\mathrm{of}\;\mathrm{calibrator}\;-\;\mathrm{OD}\;\mathrm{of}\;\mathrm{negative}\;\mathrm{control}}$$

Using this formula and the three sera from patients with IBS, neuropsychiatric, neurodegenerative, and neuroautoimmune disorders used in the assay as positive controls gave an index greater than 2.0, which is considered positive, while the background of the ELISA assay was not more than 0.150 D.

### 4.5. Statistical Analysis

Wilcoxon test was used to determine whether the antibody levels differed significantly between groups. The antibodies that were found significant with *p*-values below 0.05 were then tested for FDR for multiple-hypothesis testing. Only antibodies with FDR <0.1 were considered statistically significant. The same tests were used to compare the control group to the different severities of COVID-19 patients.

Box plots comparing the different levels of autoantibodies from COVID-19 patients (mild, severe, and oxygen-dependent groups) and healthy controls were generated using the R version 4.1 (The R Project for Statistical Computing. https://www.r-project.org/, accessed on 9 August 2021), R studio Version 1.4.1717 (R-Studio. https://www.rstudio.com/, accessed on 9 August 2021), and the R packages ggpubr, ggplot2, dplyr, datatable and gridExtra. The same plots were created comparing all the COVID-19 with the healthy controls.

Mann-Whitney U effect size was calculated using IBM SPSS Statistics software, version 27. Spearman’s rho correlation coefficient between all the different antibody levels was calculated using the same SPSS software.

## Figures and Tables

**Table 1:** Dysregulated levels of autoantibodies, whether elevated or lower, were observed throughout with severity of COVID-19 illness. The table above presents the effect size between the two groups. The color of the cells depicts the comparisons of the means of the latitudinal group relative to the longitudinal group. Antibodies against glial fibrillary acidic protein and glutamate receptors were omitted from this table because their levels were not observed to be dysregulated in COVID-19 patients.

Antibody	Severity		Covid	Mild	Severe	Oxygen		Covid	Mild	Severe	Oxygen
**Glutamic Acid Decarboxylase (GAD-65)**	**Control**	**IgA**	0.05	0.05	0.11	0.14	**IgG**	0.28	0.38	0.30	0.05
	**Mild**		-	-	0.17	0.18		-	-	0.13	0.31
	**Severe**		-	-	-	0.03		-	-	-	0.22
**Acetycholine Receptor**	**Control**	**IgA**	0.24	0.21	0.28	0.22	**IgG**	0.01	0.01	0.03	0.01
	**Mild**		-	-	0.10	0.04		-	-	0.03	0.01
	**Severe**		-	-	-	0.05		-	-	-	0.06
**α-Synucleins**	**Control**	**IgA**	0.31	0.34	0.33	0.24	**IgG**	0.04	0.12	0.06	0.06
	**Mild**		-	-	0.07	0.01		-	-	0.16	0.01
	**Severe**		-	-	-	0.05		-	-	-	0.11
**Amyloid β Peptide**	**Control**	**IgA**	0.29	0.35	0.28	0.18	**IgG**	0.21	0.22	0.29	0.09
	**Mild**		-	-	0.00	0.07		-	-	0.13	0.10
	**Severe**		-	-	-	0.06		-	-	-	0.20
**Brain Derived Neurotrophic Factor**	**Control**	**IgA**	0.10	0.07	0.13	0.05	**IgG**	0.25	0.16	0.29	0.45
	**Mild**		-	-	0.05	0.00		-	-	0.14	0.32
	**Severe**		-	-	-	0.04		-	-	-	0.20
**Cerebellar**	**Control**	**IgA**	0.01	0.05	0.01	0.12	**IgG**	0.17	0.11	0.27	0.13
	**Mild**		-	-	0.05	0.15		-	-	0.20	0.05
	**Severe**		-	-	-	0.08		-	-	-	0.14
**D1 Receptor**	**Control**	**IgA**	0.12	0.11	0.16	0.07	**IgG**	0.02	0.12	0.08	0.27
	**Mild**		-	-	0.06	0.01		-	-	0.20	0.41
	**Severe**		-	-	-	0.06		-	-	-	0.22
**D2 Receptor**	**Control**	**IgA**	0.23	0.26	0.22	0.16	**IgG**	0.14	0.22	0.13	0.01
	**Mild**		-	-	0.00	0.06		-	-	0.10	0.23
	**Severe**		-	-	-	0.04		-	-	-	0.15
**Enteric Nerve**	**Control**	**IgA**	0.10	0.10	0.11	0.04	**IgG**	0.23	0.25	0.28	0.14
	**Mild**		-	-	0.02	0.03		-	-	0.07	0.08
	**Severe**		-	-	-	0.04		-	-	-	0.14
**Ganglioside**	**Control**	**IgA**	0.02	0.02	0.05	0.09	**IgG**	0.13	0.25	0.05	0.05
	**Mild**		-	-	0.03	0.12		-	-	0.21	0.20
	**Severe**		-	-	-	0.12		-	-	-	0.00
**Myelin Basic Protein**	**Control**	**IgA**	0.23	0.22	0.25	0.22	**IgG**	0.05	0.01	0.02	0.14
	**Mild**		-	-	0.04	0.02		-	-	0.02	0.14
	**Severe**		-	-	-	0.02		-	-	-	0.14
**Myelin Oligodendrocyte Glycoprotein**	**Control**	**IgA**	0.09	0.00	0.14	0.15	**IgG**	0.04	0.08	0.04	0.32
	**Mild**		-	-	0.15	0.18		-	-	0.14	0.40
	**Severe**		-	-	-	0.04		-	-	-	0.30
**NMDA Receptor**	**Control**	**IgA**	0.22	0.28	0.23	0.20	**IgG**	0.46	0.39	0.51	0.63
	**Mild**		-	-	0.01	0.01		-	-	0.18	0.46
	**Severe**		-	-	-	0.02		-	-	-	0.30
**S100-B**	**Control**	**IgA**	0.03	0.02	0.06	0.08	**IgG**	0.31	0.28	0.38	0.31
	**Mild**		-	-	0.05	0.09		-	-	0.16	0.05
	**Severe**		-	-	-	0.11		-	-	-	0.09
**Tau Protein**	**Control**	**IgA**	0.15	0.22	0.13	0.01	**IgG**	0.28	0.36	0.30	0.17
	**Mild**		-	-	0.05	0.17		-	-	0.04	0.19
	**Severe**		-	-	-	0.11		-	-	-	0.15
**Key:** Effect Size	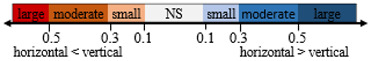										

**Table 2:** Correlation Between Antibody Levels. Spearman’s rho correlation coefficients showed very strong correlations between all IgA antibody levels. * Correlation is significant at the 0.05 level (2-tailed). ** Correlation is significant at the 0.01 level (2-tailed).

	**Acetycholine Receptor**	**Amyloid** **β** **Peptide**	**α-** **Synucleins**	**BDNF**	**Cerebellar**	**D1 Receptor**	**D2 Receptor**	**Enteric nerve**	**Ganglioside**	**Myelin Basic Protein**	**Myelin Oligodendrocyte Glycoprotein**	**NMDA Receptor**	**S100B**	**Tau Protein**	**GAD65**
**SARS CoV 2 IgG**	0.033	−0.118	0.064	0.341 **	−0.121	0.164 *	−0.001	−0.108	0.077	0.098	0.173 **	0.504 **	−0.223 **	−0.133 *	−0.093
**Acetycholine receptor**	1	0.801 **	0.795 **	0.713 **	0.809 **	0.762 **	0.808 **	0.793 **	0.787 **	0.582 **	0.762 **	0.571 **	0.721 **	0.731 **	0.757 **
**Amyloid** **β** **peptide**	0.943 **	1	0.811 **	0.644 **	0.882 **	0.823 **	0.849 **	0.836 **	0.755 **	0.546 **	0.739 **	0.552 **	0.758 **	0.765 **	0.845 **
**α-** **synucleins**	0.954 **	0.953 **	1	0.721 **	0.874 **	0.775 **	0.819 **	0.754 **	0.844 **	0.572 **	0.744 **	0.643 **	0.648 **	0.671 **	0.724 **
**BDNF**	0.930 **	0.896 **	0.912 **	1	0.659 **	0.762 **	0.702 **	0.608 **	0.689 **	0.540 **	0.711 **	0.714 **	0.487 **	0.555 **	0.629 **
**Cerebellar**	0.894 **	0.891 **	0.894 **	0.916 **	1	0.765 **	0.818 **	0.816 **	0.815 **	0.521 **	0.735 **	0.522 **	0.736 **	0.724 **	0.828 **
**D1 Receptor**	0.934 **	0.922 **	0.907 **	0.934 **	0.915 **	1	0.836 **	0.788 **	0.729 **	0.616 **	0.822 **	0.686 **	0.685 **	0.748 **	0.806 **
**D2 Receptor**	0.959 **	0.949 **	0.960 **	0.931 **	0.918 **	0.923 **	1	0.834 **	0.744 **	0.606 **	0.776 **	0.582 **	0.693 **	0.797 **	0.853 **
**Enteric nerve**	0.918 **	0.893 **	0.891 **	0.893 **	0.900 **	0.907 **	0.903 **	1	0.705 **	0.541 **	0.729 **	0.536 **	0.728 **	0.762 **	0.801 **
**Ganglioside**	0.895 **	0.874 **	0.891 **	0.932 **	0.934 **	0.917 **	0.915 **	0.884 **	1	0.593 **	0.719 **	0.673 **	0.647 **	0.620 **	0.631 **
**Myelin Basic Protein**	0.796 **	0.772 **	0.777 **	0.765 **	0.743 **	0.721 **	0.768 **	0.721 **	0.708 **	1	0.711 **	0.556 **	0.650 **	0.742 **	0.479 **
**Myelin Oligodendrocyte Glycoprotein**	0.850 **	0.835 **	0.820 **	0.842 **	0.832 **	0.842 **	0.833 **	0.831 **	0.837 **	0.819 **	1	0.721 **	0.720 **	0.751 **	0.723 **
**NMDA Receptor**	0.792 **	0.741 **	0.738 **	0.824 **	0.869 **	0.799 **	0.787 **	0.816 **	0.868 **	0.732 **	0.844 **	1	0.392 **	0.478 **	0.442 **
**S100B**	0.835 **	0.833 **	0.802 **	0.852 **	0.856 **	0.855 **	0.816 **	0.830 **	0.833 **	0.849 **	0.896 **	0.847 **	1	0.804 **	0.673 **
**Tau Protein**	0.866 **	0.872 **	0.848 **	0.851 **	0.877 **	0.829 **	0.859 **	0.833 **	0.834 **	0.900 **	0.874 **	0.836 **	0.920 **	1	0.761 **
**GAD65**	0.913 **	0.883 **	0.885 **	0.906 **	0.897 **	0.917 **	0.902 **	0.910 **	0.898 **	0.730 **	0.885 **	0.866 **	0.836 **	0.826 **	1
**Key:**	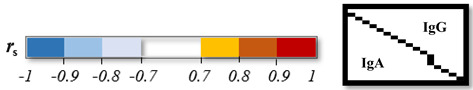														

**Table 3:** Comparison of the demographic data across the groups. * Mean interval of days between symptom onset and sera sample collection.

		Mean Age	Female		Male	
	**Control**	40.18	26/77	33.77%	51/77	66.23%
	**COVID-19**	43.8	55/169	32.54%	113/169	66.86%
**Severity**	Mean interval *	**Mean Age**	**Female**		**Male**	
**Mild**	54	32.4	28/73	38.36%	45/73	61.64%
**Severe**	60	51.49	20/64	31.25%	44/64	68.75%
**Oxygen**	59	53.84	8/32	25.00%	24/32	75.00%

## Data Availability

Data not available due to privacy and ethical concerns. There are ethical restrictions from the IRB perspective as we do not have permissions disclosed and there was sensitivity within the community cohort about not wanting any data disseminated owing to possible risk of re-identification. This was approved by the Advarra IRB (email contact: cirbi@advarra.com).

## Supplementary Materials

The following supporting information can be downloaded at: https://www.mdpi.com/article/10.3390/diagnostics13040687/s1. Table S1: Autoantigens recognized by autoantibodies in convalescing COVID-19 patients; Table S2: Dysregulated levels of autoantibodies in convalescing COVID-19 patients.
