# Supplementary material for: Dysregulated Levels of Circulating Autoantibodies against Neuronal and Nervous System Autoantigens in COVID-19 Patients
Source: Diagnostics (Basel). 2023 Feb 12;13(4):687. doi: 10.3390/diagnostics13040687 (PMC9955917; doi:10.3390/diagnostics13040687)
Supplement: Supplementary file 1 [file diagnostics-13-00687-s001.zip › diagnostics-2104228-supplementary.pdf]

Supplementary Table S1

Autoantigens Recognized by Autoantibodies in Convalescing COVID-19 Patients

	Abbreviation	Location	Function
acetylcholine receptor		Neurons, stimulated T lymphocytes	affects cognition, emotion, locomotor activity, regulation of hunger and satiety and endocrine system, linked to the negative regulation of the immune response.
glutamate receptor		Neuronal and glial cells	Excitatory neurotransmitter
amyloid β peptide			Alzheimer patients' brain have plaques of amyloid β
α-synucleins		expressed in the central and enteric nervous systems	α-Synucleins are found in healthy people, but lower titers of the antibodies were observed in patients with Parkinson's disease or multiple system atrophy
dopamine 1	D1	neurons of the central nervous system	memory, attention, impulse control, regulation of renal function, locomotion
dopamine 2	D2	Neurons in the striatum and expressed in T and B lymphocytes	locomotion, attention, sleep, memory, learning
tau protein		Neurons	Microtubule assembly, antibodies against Tau protein are lower in Alzheimer patients
Glutamic Acid Decarboxylase 65	GAD-65	Nerve terminals in GABA producing neurons and they are also expressed in the insulin-producing β-cells of the pancreas	GABA synthesis

N-methyl D-aspartate receptor	NMDA receptor	Neuronal dendrites	synaptic plasticity - mediating learning and memory
Brain Derived Neurotrophic Factor	BDNF	Brain, kidneys, prostate, motor neurons, and skeletal muscles	Long term memory storage
mitochondrial antigen	MITO		
Ganglioside		highly abundant in the nervous system	carry most of the sialic acid residues in the brain
myelin basic protein		CNS	production of myelin
myelin oligodendrocyte glycoprotein		myelin sheaths in the central nervous system	associated with different kinds of inflammatory demyelinating diseases
S100-B		expressed by astrocytes	marker of glial or ganglial cell damage

Supplementary Table S2

<u>Dysregulated Levels of Autoantibodies in Convalescing COVID-19 Patients</u>

Antibody		Severity		Covid	Mild	Severe	Oxygen			Covid	Mild	Severe	Oxygen
Glutamic Acid Decarboxylase (GAD-65)	IgA		ẍ±sd	0.9356±0.44	0.8366±0.33	1.0034±0.49	1.0388±0.52	IgG	ẍ±sd	0.8527±0.38	0.7762±0.35	0.8512±0.36	1.0496±0.42
		Control	0.9016±0.4	NS	NS	NS	NS		1.0849±0.41	****	****	***	NS
		Mild	0.8366±0.33	-	-	NS	NS		0.7762±0.35	-	-	NS	**
		Severe	1.0034±0.49	-	-	-	NS		0.8512±0.36	-	-	-	NS
Acetycholine Receptor	IgA		ẍ±sd	1.0617±0.47	0.9914±0.38	1.1235±0.52	1.1054±0.58	IgG	ẍ±sd	0.9189±0.30	0.9245±0.29	0.9084±0.3	0.9273±0.31
		Control	0.8577±0.37	***	*	**	*		0.9294±0.33	NS	NS	NS	NS
		Mild	0.9914±0.38	-	-	NS	NS		0.9245±0.29	-	-	NS	NS
		Severe	1.1235±0.52	-	-	-	NS		0.9084±0.3	-	-	-	NS
α - Synucleins	IgA		ẍ±sd	0.8664±0.43	0.8295±0.35	0.9045±0.46	0.8771±0.53	IgG	ẍ±sd	0.9585±0.34	1.0027±0.33	0.8935±0.33	0.9883±0.38
		Control	0.6202±0.31	****	****	***	*		0.9156±0.33	NS	NS	NS	NS
		Mild	0.8295±0.35	-	-	NS	NS		1.0027±0.33	-	-	NS	NS
		Severe	0.9045±0.46	-	-	-	NS		0.8935±0.33	-	-	-	NS
Amyloid β Peptide	IgA		ẍ±sd	1.1302±0.52	1.129±0.45	1.1468±0.57	1.0968±0.61	IgG	ẍ±sd	0.8775±0.30	0.883±0.27	0.8294±0.32	0.9679±0.33
		Control	0.8507±0.38	****	****	**	NS		1.0243±0.34	**	**	***	NS
		Mild	1.129±0.45	-	-	NS	NS		0.883±0.27	-	-	NS	NS

		**Severe**	1.1468±0.57	-	-	-	NS		0.8294±0.32	-	-	-	NS
**Brain Derived Neurotrophic Factor**	**IgA**		ẍ±sd	1.0663±0.53	1.0335±0.48	1.1072±0.57	1.0603±0.58	**IgG**	ẍ±sd	0.9601±0.42	0.8704±0.4	0.9737±0.41	1.1625±0.44
		**Control**	0.975±0.43	NS	NS	NS	NS		0.7365±0.31	***	NS	***	****
		**Mild**	1.0335±0.48	-	-	NS	NS		0.8704±0.4	-	-	NS	**
		**Severe**	1.1072±0.57	-	-	-	NS		0.9737±0.41	-	-	-	NS
**Cerebellar**	**IgA**		ẍ±sd	0.9879±0.48	1.0159±0.4	0.9938±0.54	0.9037±0.54	**IgG**	ẍ±sd	0.8532±0.33	0.9054±0.33	0.7808±0.32	0.8783±0.35
		**Control**	1.0017±0.44	NS	NS	NS	NS		0.997±0.41	*	NS	**	NS
		**Mild**	1.0159±0.4	-	-	NS	NS		0.9054±0.33	-	-	*	NS
		**Severe**	0.9938±0.54	-	-	-	NS		0.7808±0.32	-	-	-	NS
**D1 Receptor**	**IgA**		ẍ±sd	1.0660±0.53	1.0198±0.45	1.1196±0.58	1.0668±0.62	**IgG**	ẍ±sd	0.9092±0.36	0.7934±0.29	0.951±0.37	1.1118±0.37
		**Control**	0.9347±0.4	NS	NS	NS	NS		0.878±0.34	NS	NS	NS	**
		**Mild**	1.0198±0.45	-	-	NS	NS		0.7934±0.29	-	-	*	****
		**Severe**	1.1196±0.58	-	-	-	NS		0.951±0.37	-	-	-	NS
**D2 Receptor**	**IgA**		ẍ±sd	1.0295±0.52	1.0202±0.46	1.0518±0.58	1.0043±0.57	**IgG**	ẍ±sd	0.8362±0.36	0.7809±0.34	0.8442±0.35	0.9589±0.38
		**Control**	0.8157±0.42	***	**	*	NS		0.9548±0.4	*	**	NS	NS
		**Mild**	1.0202±0.46	-	-	NS	NS		0.7809±0.34	-	-	NS	*
		**Severe**	1.0518±0.58	-	-	-	NS		0.8442±0.35	-	-	-	NS
**Enteric Nerve**	**IgA**		ẍ±sd	0.9502±0.43	0.9302±0.35	0.9695±0.47	0.9588±0.54	**IgG**	ẍ±sd	0.8533±0.33	0.8519±0.31	0.8228±0.33	0.923±0.36
		**Control**	0.8902±0.38	NS	NS	NS	NS		1.0293±0.36	***	**	**	NS
		**Mild**	0.9302±0.35	-	-	NS	NS		0.8519±0.31	-	-	NS	NS
		**Severe**	0.9695±0.47	-	-	-	NS		0.8228±0.33	-	-	-	NS
**Ganglioside**	**IgA**		ẍ±sd	0.9936±0.48	0.9912±0.41	1.0326±0.52	0.9146±0.54	**IgG**	ẍ±sd	0.9603±0.39	1.0498±0.4	0.8897±0.37	0.8873±0.4
		**Control**	0.994±0.47	NS	NS	NS	NS		0.8445±0.37	*	**	NS	NS
		**Mild**	0.9912±0.41	-	-	NS	NS		1.0498±0.4	-	-	*	NS
		**Severe**	1.0326±0.52	-	-	-	NS		0.8897±0.37	-	-	-	NS
**Glial Fibriallary Acidic Protein**	**IgA**		ẍ±sd	1.1161±0.63	1.0755±0.56	1.2125±0.7	1.0088±0.66	**IgG**	ẍ±sd	0.9356±0.46	0.9178±0.5	0.937±0.43	0.978±0.42
		**Control**	1.0047±0.53	NS	NS	NS	NS		0.8829±0.51	NS	NS	NS	NS
		**Mild**	1.0755±0.56	-	-	NS	NS		0.9178±0.5	-	-	NS	NS
		**Severe**	1.2125±0.7	-	-	-	NS		0.937±0.43	-	-	-	NS

**Glutamate Receptor**	**IgA**		ẍ±sd	0.9005±0.44	0.9026±0.36	0.8926±0.45	0.9125±0.59	**IgG**	ẍ±sd	0.9604±0.33	0.9921±0.32	0.9388±0.33	0.9272±0.37
		**Control**	0.9206±0.4	NS	NS	NS	NS		0.9096±0.35	NS	NS	NS	NS
		**Mild**	0.9026±0.36	-	-	NS	NS		0.9921±0.32	-	-	NS	NS
		**Severe**	0.8926±0.45	-	-	-	NS		0.9388±0.33	-	-	-	NS
**Myelin Basic Protein**	**IgA**		ẍ±sd	0.9901±0.44	0.9603±0.4	1.0242±0.49	0.9916±0.45	**IgG**	ẍ±sd	0.9563±0.32	0.9399±0.33	0.9433±0.33	1.0261±0.29
		**Control**	0.7992±0.34	***	**	**	*		0.9328±0.35	NS	NS	NS	NS
		**Mild**	0.9603±0.4	-	-	NS	NS		0.9399±0.33	-	-	NS	NS
		**Severe**	1.0242±0.49	-	-	-	NS		0.9433±0.33	-	-	-	NS
**Myelin Oligodendrocyte Glycoprotein**	**IgA**		ẍ±sd	0.9284±0.43	0.8376±0.33	0.9805±0.45	1.0452±0.55	**IgG**	ẍ±sd	0.9937±0.27	0.9242±0.25	0.9933±0.26	1.1708±0.26
		**Control**	0.8533±0.35	NS	NS	NS	NS		0.9608±0.28	NS	NS	NS	**
		**Mild**	0.8376±0.33	-	-	NS	NS		0.9242±0.25	-	-	NS	****
		**Severe**	0.9805±0.45	-	-	-	NS		0.9933±0.26	-	-	-	**
**NMDA Receptor**	**IgA**		ẍ±sd	0.8570±0.39	0.8355±0.31	0.8652±0.43	0.8935±0.47	**IgG**	ẍ±sd	1.0645±0.39	0.9356±0.31	1.0829±0.4	1.3515±0.39
		**Control**	1.07±0.42	***	***	**	NS		0.6943±0.31	****	****	****	****
		**Mild**	0.8355±0.31	-	-	NS	NS		0.9356±0.31	-	-	NS	****
		**Severe**	0.8652±0.43	-	-	-	NS		1.0829±0.4	-	-	-	**
**S100-B**	**IgA**		ẍ±sd	1.0282±0.45	1.0177±0.4	1.0701±0.49	0.9637±0.51	**IgG**	ẍ±sd	0.8963±0.30	0.9306±0.29	0.8511±0.3	0.9077±0.34
		**Control**	1.0108±0.42	NS	NS	NS	NS		1.1137±0.34	****	***	****	**
		**Mild**	1.0177±0.4	-	-	NS	NS		0.9306±0.29	-	-	NS	NS
		**Severe**	1.0701±0.49	-	-	-	NS		0.8511±0.3	-	-	-	NS
**Tau Protein**	**IgA**		ẍ±sd	1.0129±0.45	1.0356±0.39	1.0219±0.49	0.9359±0.52	**IgG**	ẍ±sd	0.8439±0.28	0.8082±0.25	0.844±0.29	0.9343±0.3
		**Control**	0.889±0.39	*	**	NS	NS		1.0104±0.28	****	****	***	NS
		**Mild**	1.0356±0.39	-	-	NS	NS		0.8082±0.25	-	-	NS	NS
		**Severe**	1.0219±0.49	-	-	-	NS		0.844±0.29	-	-	-	NS

Key: ***P value*** **-** * = <0.05, **= <0.01, ***=<0.001, ****=<0.0001
